# The detrimental effect of AlGaN barrier quality on carrier dynamics in AlGaN/GaN interface

**DOI:** 10.1038/s41598-019-53732-y

**Published:** 2019-11-22

**Authors:** Žydrūnas Podlipskas, Jonas Jurkevičius, Arūnas Kadys, Saulius Miasojedovas, Tadas Malinauskas, Ramūnas Aleksiejūnas

**Affiliations:** Institute of Photonics and Nanotechnology, Vilnius University, Saulėtekio. ave. 3, Vilnius, LT-10257 Lithuania

**Keywords:** Electronic devices, Electronic properties and materials, Semiconductors, Surfaces, interfaces and thin films, Lasers, LEDs and light sources

## Abstract

Carrier recombination and scattering at the semiconductor boundaries can substantially limit the device efficiency. However, surface and interface recombination is generally neglected in the nitride-based devices. Here, we study carrier recombination and diffusivity in AlGaN/GaN/sapphire heterointerfaces with AlGaN barriers of different quality. We employ the light induced transient grating and time-resolved photoluminescence spectroscopy techniques to extract carrier lifetime in different depths of the GaN buffer as well as in the AlGaN barrier, and to evaluate the carrier diffusion coefficient in the buffer. Moreover, we assess interface recombination velocity, Shockley-Read-Hall and radiative recombination rates. We reveal the adverse barrier influence on carrier dynamics in the underlying buffer: AlGaN barrier accelerates the nonradiative carrier recombination in the GaN buffer. The interface recombination velocity in the GaN buffer increases with decreasing AlGaN barrier quality, and the dominating recombination mechanism switches from Shockley-Read-Hall to interface recombination. These phenomena are governed by a cumulative effect of various interface-deteriorating barrier defects. Meanwhile, the carrier diffusivity in the GaN buffer is not affected by the AlGaN barrier. We conclude that barrier-accelerated interface recombination can become a major carrier loss mechanism in AlGaN/GaN interface, and may substantially limit the efficiency in nitride-based UV LEDs.

## Introduction

Nonradiative surface recombination (SR) at the boundaries of a semiconductor device can be a major factor limiting the efficiency in light-emitting and laser diodes (LEDs and LDs), photovoltaic cells, and photodetectors. The detrimental effects of SR are usually tackled by adding a surface-passivating layer or, in case of a heterostructure, an interface layer. Carrier-confining interfaces have enabled a substantial increase in efficiency in arsenide- and phosphide-based photonic devices. In bare-surface GaAs the surface recombination velocity (*S*) can reach 10^7^ cm/s^1^, but a suitably engineered interface can reduce the *S* value down to 18 cm/s, as reported for AlGaAs LD heterostructures^2^. While the focus on the interface quality in arsenide- and phosphide-based photonic devices has led to tangible progress, the nonradiative recombination at the interface layer in nitride devices have been often overlooked, mainly due to the secondary role of SR in the high-efficiency InGaN LEDs. The SR velocity is generally lower in nitrides^3–7^ than in other III-V materials, and the diffusion length in the active InGaN region is relatively short^8^ due to both carrier-localizing composition fluctuations, and short carrier lifetime at high operating carrier density^9^. However, a recent theoretical study^6^ shows a noticeable impact of SR towards the efficiency of a nitride-based LED structure, and the negative impact of SR is predicted to be even higher for AlGaN-based UV LEDs or nanostructured photonic devices.

In this work, we observe and investigate an unconventional trend in AlGaN/GaN interfaces: the adverse influence of the AlGaN barrier on the interface recombination in the underlying GaN buffer. The effects of decreasing AlGaN barrier quality on carrier diffusivity and recombination were explored by time-resolved photoluminescence spectroscopy and light-induced transient grating (LITG) techniques. The density-dependent values of carrier lifetime were obtained in different depths of the GaN buffer using different excitation wavelengths. The terms of the effective carrier lifetime (interface recombination velocity, Shockley-Read-Hall and radiative recombination rates) are evaluated.

## Methods

### Samples

Twelve c-plane AlGaN/GaN/sapphire heterostructures were grown by the metalorganic chemical vapour deposition (MOCVD) technique. The 130–300 nm thick Al_*x*_Ga_1-*x*_N barriers were grown at 1090 °C and at constant tri-methyl-aluminium (TMAl) flow rate, while the Al content was controlled by changing the tri-methyl-gallium (TMGa) flow rate. Three sample sets with different barrier Al content were obtained: *x* = (0.13, 0.2, or 0.34, estimated from the XRD spectra), with ≤10% relative variation within a sample set. Variance of the ammonia flow rates (500–5000 sl/min) during the barrier growth resulted in different barrier structural quality between the samples. All barriers were grown on identical GaN/Sapphire templates with 4 µm thick GaN buffers. XRD reciprocal space maps show all barriers to be strained (see XRD data in the Supplementary Figs I and II). The XRD-estimated dislocation density (total of edge and screw DD) shows little variation in both barriers and buffers between samples and is equal to ~ 4 × 10^9^ cm^−2^.

### Measurement methods

**Figure 1:**
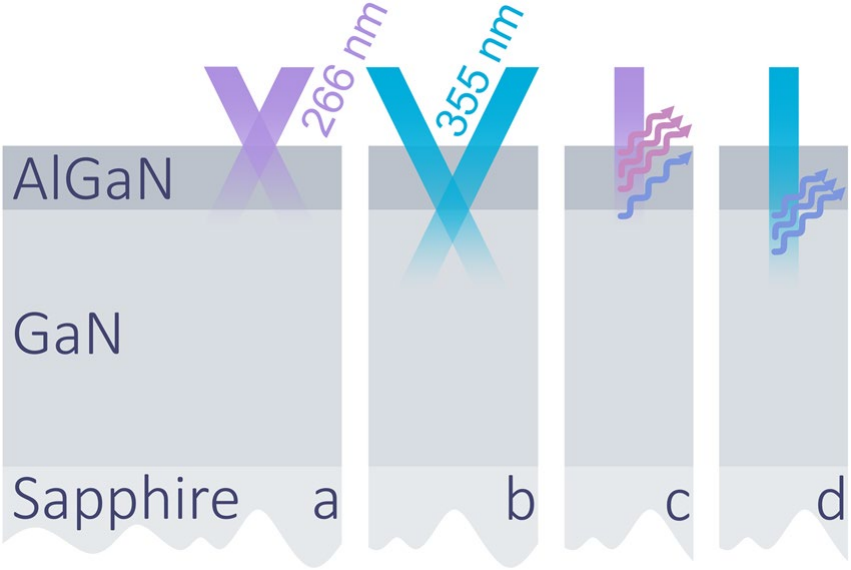
Simplified scheme of the excitation configurations: (**a**) LITG in AlGaN barrier, 266 nm excitation; (**b**) LITG in GaN buffer, 355 nm excitation; (**c**) 266 nm excitation for TRPL in the barrier and the shallow interface (initial ~50 nm in GaN); (**d**) 355 nm excitation for TRPL in the deep interface (initial ~100 nm in GaN).

Light-induced transient gratings (LITG) and time-resolved photoluminescence (TRPL) techniques were employed to investigate carrier dynamics in AlGaN barriers and GaN buffers at room temperature. The diffusion coefficient in GaN buffer was measured by LITG, whereas the carrier lifetime in GaN buffer and the AlGaN barrier was obtained from the TRPL and LITG transients. Different excitation configurations of the LITG and TRPL experiments are demonstrated in Fig. 1, where the excitation beams (*λ* = 266 nm or 355 nm, indicated) represent pulses (25 ps, 10 Hz) of an Ekspla Nd:YAG laser.

In the LITG experiment, two coherent pump pulses intersected at the angle *ϴ* on the sample creating a transient pattern of photoexcited carriers spatially modulated with spacing *Λ* = *λ*/sin*ϴ*^10^. The carrier density modulation causes a spatial modulation of the refractive index with the amplitude proportional to the photoexcited carrier density (*N*), thus recording a transient diffraction grating. The grating decay was monitored by a diffracted and delayed probe pulse in the transparency region of the sample at 1064 nm. The decay of the diffraction grating was caused by two effects: recombination of the excess carriers and their diffusion, which tends to homogenize the carrier spatial distribution. Evaluation of grating decay time (*τ*_G_) at various grating periods Λ enabled the determination of excess carrier ambipolar diffusion coefficient (*D*) and recombination time (*τ*): 1/*τ*_G_ = 1/*τ* + 4π^2^*D*/Λ^2^ ^10^. Carrier dynamics were selectively observed in the AlGaN barrier and the GaN buffer by employing excitation pulses of 266 nm or 355 nm wavelength, respectively (see Fig. 1a,b). The selective excitation of the GaN buffer is possible as the AlGaN barriers (*x* = 0.13–0.34; *E*_g_ = 3.7–4.2 eV) are transparent to the 355 nm photons (*hν* = 3.49 eV). Excess carrier density *N* (5 × 10^18^ cm^−3^–2 × 10^20^ cm^−3^) was controlled by varying the energy density of the excitation pulse (0.03–1 mJ/cm^2^).

The TRPL experiment was performed using a Hamamatsu streak camera with an Acton monochromator. Above-bandgap 266 nm excitation of both AlGaN and GaN and the available spectral resolution of the TRPL technique allowed to extract carrier lifetime in both barrier and buffer simultaneously (see Fig. 1c), while employing 355 nm excitation provided greater absorption depth in GaN (Fig. 1d). As a result, carrier recombination was investigated in different depths of the GaN buffer: in the initial ~50 nm and in the initial ~100 nm (from here on, ‘shallow’ and ‘deep’ interface, respectively). Carrier recombination was observed at excess carrier densities ranging from 1 × 10^17^ to 3 × 10^19^ cm^−3^.

The lifetime extraction from LITG- and TRPL-measured transients was carried out from the trailing transient end. The TRPL transients for buffer or barrier were obtained by integrating the full spectrum of band-to-band transitions in GaN or AlGaN, respectively. See Supplementary Material for typical LITG and TRPL transients (Supplementary Fig. III,VI) and a more detailed description of the lifetime extraction procedure.

## Results and Discussion

**Figure 2:**
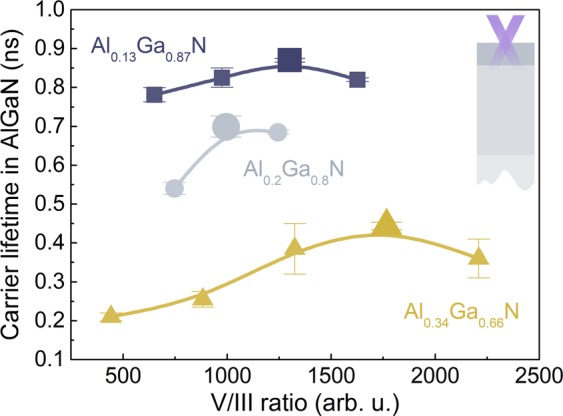
Carrier lifetime *τ*_AlGaN_ in Al_x_Ga_1-x_N barriers with different Al content *x* as a function of V/III ratio; *τ*_AlGaN_ was assessed from LITG transients recorded with pulses of *λ* = 266 nm.

Carrier lifetimes in AlGaN barriers (*τ*_AlGaN_) with various Al content (*x*) are demonstrated in Fig. 2 as a dependence on the V/III molar ratio, which was calculated from the gas flow rates: NH_3_/(TMGa + TMAl). A non-monotonous trend of initial increase and eventual decrease is observed for all sample sets and may be attributed to the different origin of the growth-related defects, discussed in the following paragraphs.

The initially low *τ*_AlGaN_ at low V/III ratio (NH_3_ flow rate) is attributed to nitrogen deficiency-related defects: N vacancy-bound defects (such as V_N_-decorated dislocations), and impurities (such as carbon occupying the N-sites). The growth of carrier lifetime with V/III ratio is due to diminishing of nitrogen deficiency and the resulting decrease in defect concentration. Increase in the NH_3_ flow rate has been shown to cause a decrease in the V_N_ concentration in GaN^11,12^, AlGaN^13,14^ and AlN^15^; a similar decrease is expected for the carbon concentration in GaN^16^.

At the highest V/III ratios the carrier lifetime decreases due to the excess-nitrogen induced point defects and degraded structural quality. Overflow of N can saturate the lattice sites and hinder the surface mobility of Ga^17^ and Al adatoms^13,18^, thus generating Ga^19^ and Al vacancies (V_Ga_, V_Al_), or deteriorating surface morphology^18,20,21^. V_Ga_ and V_Al_-related deep gap defects can be attributed to V_Ga_-O complexes^22,23^, dislocations decorated with V_Ga_-O^24–26^ or V_Al_-O complexes^27,28^.

Further discussion of the carrier lifetime *τ*_AlGaN_ concerns the samples with different Al content grown at respectively optimized V/III ratios (see larger symbols in Fig. 2). Peak carrier lifetime decreases from 0.9 to 0.4 ns in the AlGaN barriers with increasing Al content (from 0.13 to 0.34; see Fig. 2); this result is consistent with the previously reported trend in AlGaN epilayers with wide Al composition range^29^. Evidently, the optimization of growth conditions does not compensate for the decrease in structural quality with growing Al content. This decrease is typically attributed to low Al adatom mobility^30^ resulting in extended and point defects, large and fine scale lateral phase separation^31,32^ or spontaneous phase modulation^31^. It can be speculated that Al content-induced decrease in *τ*_AlGaN_ is governed by native or impurity related point defects. Chichibu *et al*. observed^33^ the increasing concentration of V_III_ (or V_III_ complexes) with increasing Al content in AlGaN, and argued^34^ that V_Ga_ are the main carrier lifetime killers in GaN. Meanwhile, studies on impurities in AlGaN demonstrate the increasing oxygen^13,16,31,35^ and carbon^16^ concentrations with increasing Al content.

While a definitive interpretation of mechanisms governing *τ*_AlGaN_ at different growth conditions and barrier compositions is unavailable, it can be stated that the nonradiative carrier recombination in the studied samples is driven by defects of numerous origins.

**Figure 3:**
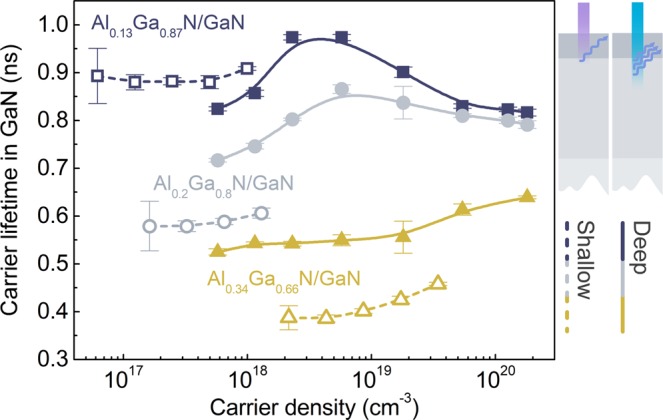
Carrier lifetime *τ*_GaN_ in GaN buffers with Al_x_Ga_1-x_N barriers of various composition as a function of photoexcited carrier density; *τ*_GaN_ was assessed at the shallow and deep interface of the AlGaN/GaN heterostructures using excitation pulses of 266 nm and 355 nm wavelength, respectively.

The data on carrier recombination at different AlGaN/GaN interfaces is presented in Fig. 3 as carrier lifetime in GaN buffer (*τ*_GaN_) dependences on carrier density; the lifetime curves for shallow and deep interface excitations are depicted as dashed and continuous lines, respectively. The rise-and-fall behaviour of carrier lifetime can be attributed to the competition between the nonradiative and radiative terms: the initial saturation of the nonradiative channel and the eventual emergence of the radiative term with increasing *N*. The nonradiative recombination may include contributions from Shockley-Read-Hall (SRH) and interface recombination channels. The SRH channel saturation and lifetime increase was previously observed in synthetic diamonds^36^, where defects acting as centres of nonradiative recombination were saturated at high photoexcitation levels.

The dominance of either SRH or interface recombination channel can be determined by varying the excitation wavelength^37,38^. In this study, this is achieved by measuring carrier lifetime in shallow and deep interface.

In GaN buffer with Al_0.13_Ga_0.87_N barrier the lifetime *τ*_GaN_ increases by switching from deep to shallow interface (see blue lines in Fig. 3). This is an indication that the defect concentration is decreasing with increasing effective layer thickness (decreasing depth), and that the interface has a minor impact on carrier recombination. A similar carrier lifetime increase with increasing layer thickness occurs in bulk GaN due to a decrease in dislocation density^39–43^.

The opposite case is observed in the heterostructures with higher Al content (see gray and yellow lines for *x* = 0.2 and 0.34, respectively). Here, switching to the shallow interface results in a decrease in the carrier lifetime *τ*_GaN_. Moreover, the ratio between the deep and the shallow interface lifetimes *τ*_GaN_ increases with the barrier Al content: from 1.2 (*x* = 0.2) to 1.4 (*x* = 0.34) at the lowest carrier density. These trends indicate the increasing role of the interface recombination channel.

The most important and non-intuitive feature of the carrier recombination is the considerable change in the *τ*_GaN_(*N*) curves (seen in Fig. 3) with the increasing Al content in the AlGaN barrier. As the Al content increases from 13% to 34%, the lifetime in the GaN buffer decreases by a factor of ~2 (calculated as the average shallow interface *τ*_GaN_). Additionally, the onset of the increase in *τ*_GaN_(*N*) shifts to higher carrier densities. At first glance, the Al content in the AlGaN barrier has a direct effect on the carrier recombination in GaN buffer.

**Figure 4:**
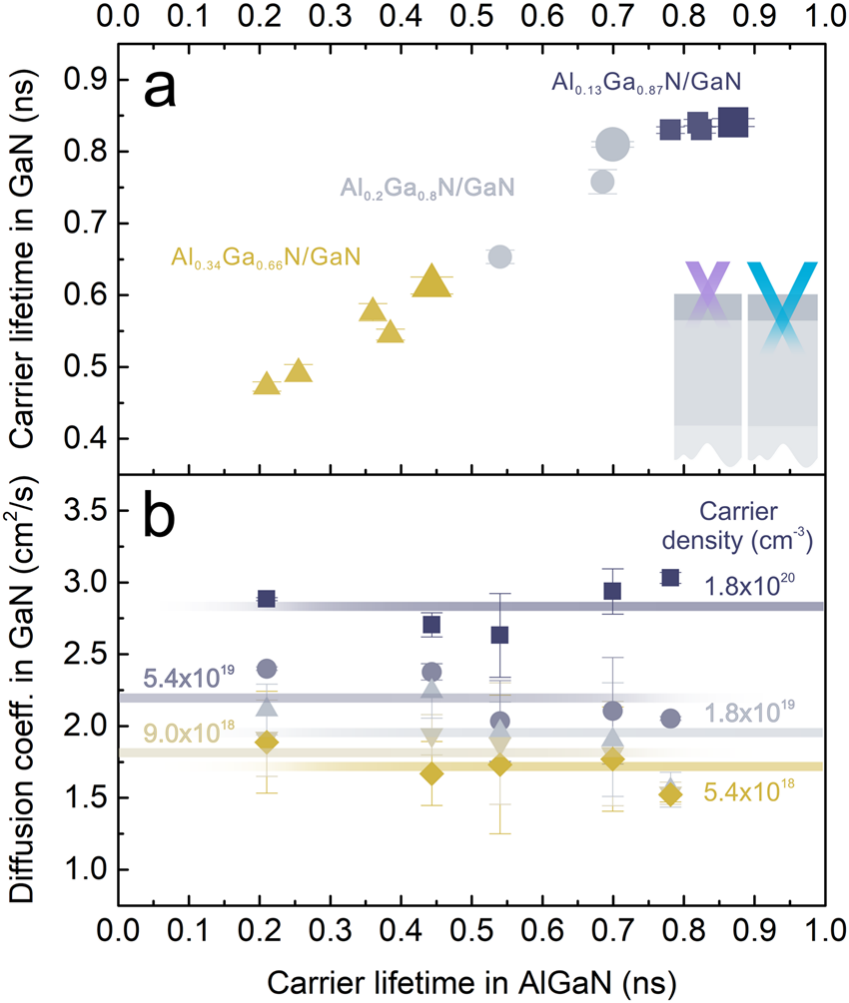
(**a**) Carrier lifetime *τ*_GaN_ at fixed carrier density *N* = 5.4 × 10^19^ cm^−3^; (**b**) diffusion coefficient *D*_GaN_ at various *N* in GaN buffers as a function of carrier lifetime *τ*_AlGaN_ in AlGaN barriers; *τ*_GaN_, *D*_GaN_ were assessed from the LITG transients recorded with pulses of 355 nm wavelength, while *τ*_AlGaN_ – with pulses of 266 nm wavelength.

To determine if the variation in lifetime *τ*_GaN_ between the samples is related to the barrier composition only, deep interface *τ*_GaN_ was assessed additionally in samples with different defect origins (each sample corresponding to one point in Fig. 2). The relation between *τ*_GaN_ and *τ*_AlGaN_ is demonstrated in Fig. 4a, where *τ*_GaN_ scales linearly with *τ*_AlGaN_ for all samples. This correlation also holds for samples of same barrier composition and different structural quality (symbols of matching color in Fig. 4a). Apparently, the carrier lifetime decrease in GaN cannot be attributed to a particular barrier defects and is controlled by the overall AlGaN barrier quality.

The effects of barrier quality on carrier scattering in GaN were assessed by the diffusion coefficient *D*_GaN_ measurements; the values of *D*_GaN_ as a function of *τ*_AlGaN_ at various carrier densities *N* in GaN are demonstrated in Fig. 4b. No dependence of carrier diffusivity on barrier lifetime *τ*_AlGaN_ was observed throughout the explored *N* range (from 5.4 × 10^18^ to 1.8 × 10^20^ cm^−3^). The lack of such dependence in the studied GaN buffers with different AlGaN barriers indicate the absence of barrier effect on carrier scattering in GaN, contrary to the influence on carrier lifetime *τ*_GaN_. It could be speculated that the carrier diffusivity in GaN is adversely affected by the AlGaN barrier, but any measurable change is hidden behind the dominating scattering mechanism in room temperature GaN (carrier - optical phonon).

The average *D*_GaN_ value depends on carrier density and increases from 1.7 to 2.8 cm^2^/s with increasing *N*. Similar diffusion coefficients at comparable carrier densities are reported for thick high-quality HVPE-grown GaN^44^, where increase in carrier diffusivity with *N* was attributed to carrier degeneracy^44^. Degeneracy could also play a role in masking any adverse effects of AlGaN barrier on carrier scattering in GaN buffer.

The extracted *D*_GaN_ values are required for a more detailed analysis of diffusion-limited interface recombination pathway at the AlGaN/GaN interface. The interface recombination velocity (*S*_i_) was assessed by fitting LITG transients with the following model:

1 $$\frac{\partial N(x,z,t)}{\partial t}=\nabla [{D}_{{\rm{GaN}}}\nabla N(x,z,t)]-\frac{N(x,z,t)}{{\tau }_{{\rm{GaN}}}^{{\rm{SRH}}}}-{B}_{{\rm{GaN}}}{N}^{2}(x,z,t)+G(x,z,t)$$

$${\frac{\partial N(x,z,t)}{\partial z}|}_{z=0}=\frac{{S}_{{\rm{i}}}N(x,0,t)}{{D}_{{\rm{GaN}}}}$$

$${\frac{\partial N(x,z,t)}{\partial z}|}_{z=d}=0$$

where ${\tau }_{{\rm{GaN}}}^{{\rm{SRH}}}$ – SRH lifetime in GaN, *B*_GaN_ – radiative recombination coefficient in GaN, *G*(*x, z, t*) – spatially modulated carrier generation rate in GaN, and *d* – the GaN buffer thickness. The radiative recombination coefficient *B*_GaN_ is considered as identical between the buffers. The *B*_GaN_ value was calculated in the sample with the highest quality barrier and equals to 1.5 × 10^−11^ cm^3^/s, which is close to the previously reported values in thick ELO- and HVPE-grown GaN layers^44,45^.

**Figure 5:**
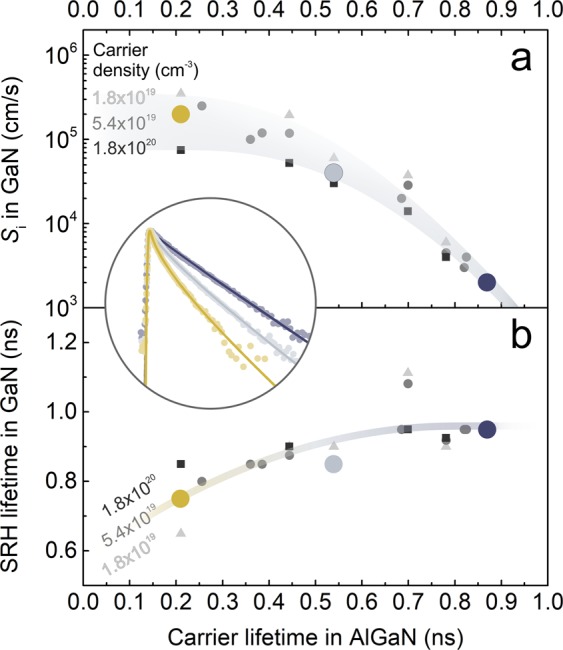
Interface recombination velocity *S*_i_ (**a**) and SRH carrier lifetime ${\tau }_{{\rm{GaN}}}^{{\rm{SRH}}}$ (**b**) at various carrier densities *N* in GaN buffers as a function of carrier lifetime *τ*_AlGaN_ in AlGaN barriers; the *S*_i_ and ${\tau }_{{\rm{GaN}}}^{{\rm{SRH}}}$ values signified as larger colored circles are extracted from the LITG transients demonstrated in the inset; inset transients (points) were recorded with pulses of 355 nm at *N* = 5.4 × 10^19^ cm^−3^ in GaN buffers; inset lines denote fitting with the (1) model.

Three LITG transients recorded at *N* = 5.4 × 10^19^ cm^−3^ in GaN buffers with various AlGaN barrier quality are depicted with fits (according to Eq. 1) in the inset of Fig. 5; the corresponding *S*_i_ and ${\tau }_{{\rm{GaN}}}^{{\rm{SRH}}}$ values are illustrated as larger coloured circles in Fig. 5a,b. These values (and the fitting results for all samples and carrier densities) are shown as a function of the carrier lifetime in the AlGaN barrier *τ*_AlGaN_. A strong *S*_i_ dependence on barrier quality is observed: *S*_i_ increases from 2 × 10^3^ to 2 × 10^5^ cm/s as *τ*_AlGaN_ decreases from 0.9 to 0.2 ns at *N* = 5.4 × 10^19^ cm^−3^ (circles in Fig. 5a). Compared to bare-surface GaN layers, where surface recombination velocity *S* typically reaches 1–5 × 10^4^ cm/s^3–5,46^, buffers with high quality barriers (*τ*_AlGaN_ > 0.7 ns) display improved boundary properties. Such improvement is observed in a wide range of III/V and II/VI material interfaces, e.g., *S* ≈ 1 × 10^7^ cm/s in bare-surface GaAs^1^ is greatly reduced with *p*^+^ GaAs layer (*S*_i_ = 1.5 × 10^4^ cm/s)^37^, Ga_2_O_3_ layer (*S*_i_ ≈ 4.5 × 10^3^ cm/s)^1^, GaAs/AlAs superlattice (*S*_i_ = 40 cm/s)^47^, AlGaAs layer (*S*_i_ = 18 cm/s)^2^, or GaInP layer (*S*_i_ = 1.5 cm/s)^48^. However, the GaN buffers with moderate and low AlGaN barrier quality (*τ*_AlGaN_ < 0.7 ns) suffer a substantial decrease in interface quality (Fig. 5a). This trend has not been reported for nitride interfaces.

Interface recombination velocity depends not only on the interface (barrier) quality but also on the carrier density. In the buffer with low quality barrier (*τ*_AlGaN_ ≈ 0.2 ns), *S*_i_ gradually decreases from 3.5 × 10^5^ to 7.5 × 10^4^ cm/s with *N* increasing from 1.8 × 10^19^ to 1.8 × 10^20^ cm^−3^. However, increase in barrier quality weakens this dependence (see Fig. 5a). According to our calculations, the discussed carrier density range is sufficient for screening of the electric polarization field in the interface. Therefore, density-driven *S*_i_ decrease was attributed to the saturation of interface recombination channel, mirroring the *τ*_GaN_(*N*) dependences discussed in the section of Fig. 3.

Compared to the *S*_i_ dependence on *τ*_AlGaN_, the impact of barrier quality on the ${\tau }_{{\rm{GaN}}}^{{\rm{SRH}}}$ term is diminished: ${\tau }_{{\rm{GaN}}}^{{\rm{SRH}}}$ features a ~20% decrease as *τ*_AlGaN_ decreases from 0.9 to 0.2 ns at *N* = 5.4 × 10^19^ cm^−3^ (circles in Fig. 5b). Meanwhile, growing carrier density *N* causes a ~30% increase in the lifetime ${\tau }_{{\rm{GaN}}}^{{\rm{SRH}}}$ in the buffer with low quality barrier (*τ*_AlGaN_ ≈ 0.2 ns), but has no perceptible effect on ${\tau }_{{\rm{GaN}}}^{{\rm{SRH}}}$(*N*) dependence in buffers with higher quality barriers.

The depth-wise expansion of the barrier influence is implied by the shifting balance between the volume- and interface-bound recombination channels (characterized with ${\tau }_{{\rm{GaN}}}^{{\rm{SRH}}}$ and *S*_i_, respectively). Moderate and high quality samples (*τ*_AlGaN_ > 0.4 ns) show little to no change in ${\tau }_{{\rm{GaN}}}^{{\rm{SRH}}}$, while demonstrating significant variation in *S*_i_ (see respective dependences on *τ*_AlGaN_ in Fig. 5a,b), pointing to an interface-bound defective area. The expansion of the barrier influence for the low quality samples (*τ*_AlGaN_ < 0.4 ns) is observed as a decrease in ${\tau }_{{\rm{GaN}}}^{{\rm{SRH}}}$ with decreasing *τ*_AlGaN_. A mechanism of quality deterioration for the GaN buffer volume may be attributed to the diffusion of interface-degrading impurities such as carbon and oxygen or point defects such as V_N_ and V_Ga_ from the barrier (or the interface) to the buffer volume. For instance, multidirectional diffusion of V_N_ and V_Ga_ in GaN is expected at MOCVD-characteristic temperatures^49,50^. Such speculation is further supported by the saturation of the *S*_i_ increase with decreasing *τ*_AlGaN_ paired with the simultaneous and accelerating decrease in ${\tau }_{{\rm{GaN}}}^{{\rm{SRH}}}$ (see Fig. 5): this juxtaposition implies a possible defect redistribution from the interface to the volume.

To finalize the characterization of the interface recombination, we provide an insight into the relation between the recombination velocity *S*_i_ and the internal quantum efficiency (IQE). For a model SQW heterostructure with one electrically active interface, the IQE can be roughly estimated using a simple rate equation^3^:

2 $${\rm{IQE}}=\frac{BN}{BN+1/{\tau }^{{\rm{SRH}}}+{S}_{{\rm{i}}}/d}$$

where *d* is the thickness of the active region. Following this model, the IQE in a *d* = 5 nm SQW (AlGaN/GaN/AlGaN) drops by two orders of magnitude with the decrease in interface quality (*S*_i_ increase from 2 × 10^3^ to 2 × 10^5^ cm/s) at *N* = 1.8 × 10^19^ cm^−3^. In comparison, the adverse impact of surface recombination at the bare-surface areas of an InGaN based LED has been previously theoretically evaluated at 5–7% of the device wall-plug efficiency, and predicted to be even stronger in UV LEDs^6^. We show that in AlGaN/GaN heterostructures the drop in the IQE may be significantly higher due to carrier recombination at the heterointerfaces.

In conclusion, we study carrier dynamics in AlGaN/GaN heterointerfaces with numerous defect origins in AlGaN barriers, grown on identical GaN buffers. We demonstrate that the barrier alters and governs the carrier dynamics in the underlying buffer: carrier lifetime in the GaN buffer decreases with the AlGaN barrier quality. The majority of photo-generated carriers in the affected GaN buffers recombine close to the heterointerface, and the dominating decay mechanism is attributed to interface recombination. The interface recombination velocity increases from low-10^3^ to mid-10^5^ cm/s with decreasing barrier quality. The carrier recombination in the GaN buffer is not governed by a particular type of defect in the AlGaN barrier, and is controlled by the overall barrier quality. Contrary to the influence on carrier recombination rate, the AlGaN barrier has no observable effect on the carrier scattering and diffusivity in the GaN buffer. Finally, we show that the interface recombination, as a major carrier loss mechanism, may substantially limit IQE in nitride-based UV LEDs.

## Supplementary information

Supplementary information

## Data Availability

All data analysed during this study are included in this published article.
