# Supplementary material for: The detrimental effect of AlGaN barrier quality on carrier dynamics in AlGaN/GaN interface
Source: Sci Rep. 2019 Nov 22;9:17346. doi: 10.1038/s41598-019-53732-y (PMC6874540; doi:10.1038/s41598-019-53732-y)
Supplement: Supplementary file 1 — Supplementary information [file 41598_2019_53732_MOESM1_ESM.pdf]

Supplementary Material

The Detrimental Effect of AlGaN Barrier Quality on Carrier Dynamics in AlGaN/GaN Interface

Žydrūnas Podlipskas, Jonas Jurkevičius, Arūnas Kadys, Saulius Miasojedovas, Tadas Malinauskas, and Ramūnas Aleksiejūnas

Institute of Photonics and Nanotechnology, Vilnius University, Saulėtekio. ave. 3, Vilnius, LT-10257, Lithuania

1. XRD DATA

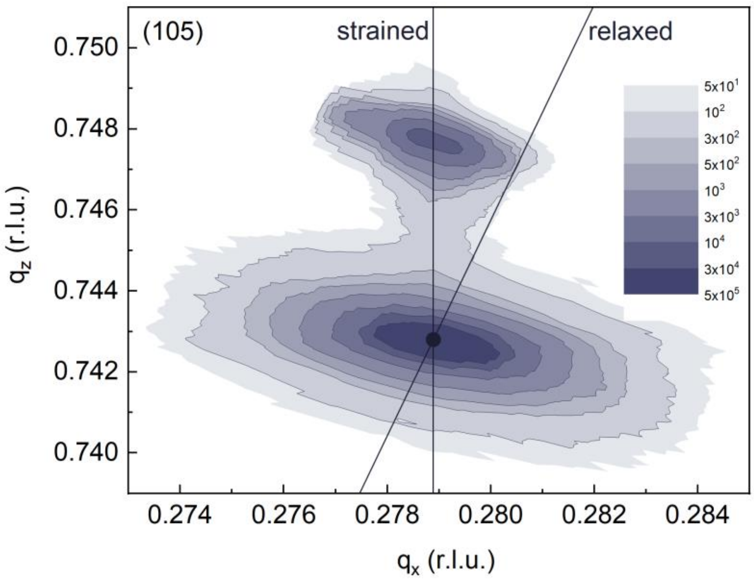

Fig. I. High-resolution XRD reciprocal space map of one of the $Al_{0.13}Ga_{0.67}N$ barriers measured at (105) reflection. Lines indicate positions of lattice constants in a relaxed or strained AlGaN layer on a GaN surface. This image is typical and representative of all three sample sets.

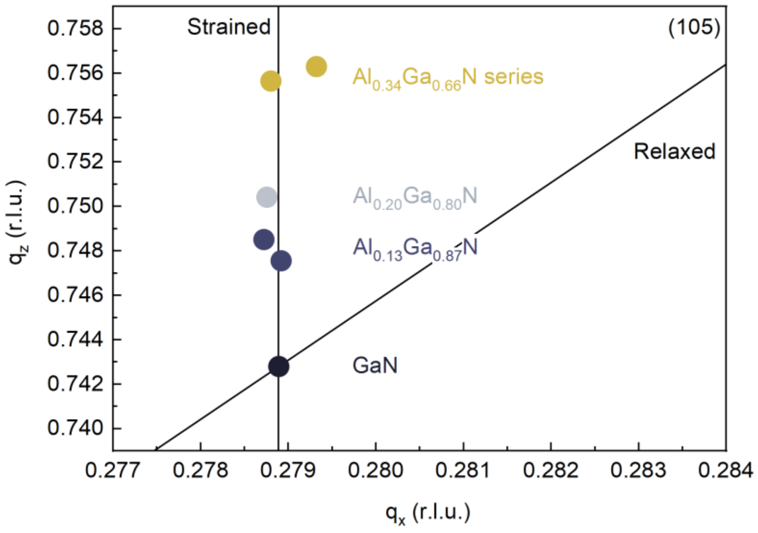

Fig. II. HR-XRD RSM peak positions for different AlGaN barriers with different composition (noted). Lines indicate positions of lattice constants in a relaxed or strained AlGaN layer on a GaN surface.

2. LITG/TRPL TRANSIENTS AND CARRIER LIFETIME EXCTRACTION

The LITG- and TRPL- measured transients for AlGaN barriers and GaN buffers are multi-exponential (see figures III-VI). As the initial (fast) part of the transients is governed by numerous density-dependent processes, all lifetime values were extracted from the less ambiguous trailing end of the transients, where a single-exponential fit (noted by solid lines) can be obtained. The mechanisms behind the non-linearity are different for AlGaN and GaN.

In case of AlGaN barriers (see figures III and V), the initial (fast) part of the transient is governed by the density-dependent recombination (e.g., radiative) of free/weakly localized carriers. Meanwhile, the trailing-end (slow) is mainly governed by Shockley-Read-Hall recombination of localized carriers.[1]

In case of GaN buffers (see figures IV and VI), where no carrier localization is present, the initial part of transients is governed by the radiative and interface recombination terms, while the trailing-end is governed by the SRH term.

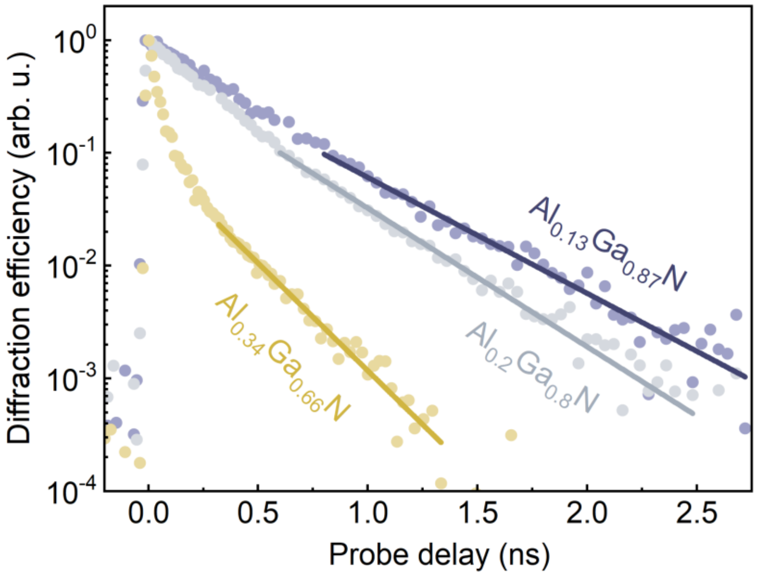

Fig. III. LITG transients for three AlGaN barriers (one from each sample set) recorded with 266 nm pulses at 0.3 mJ/cm² excitation.

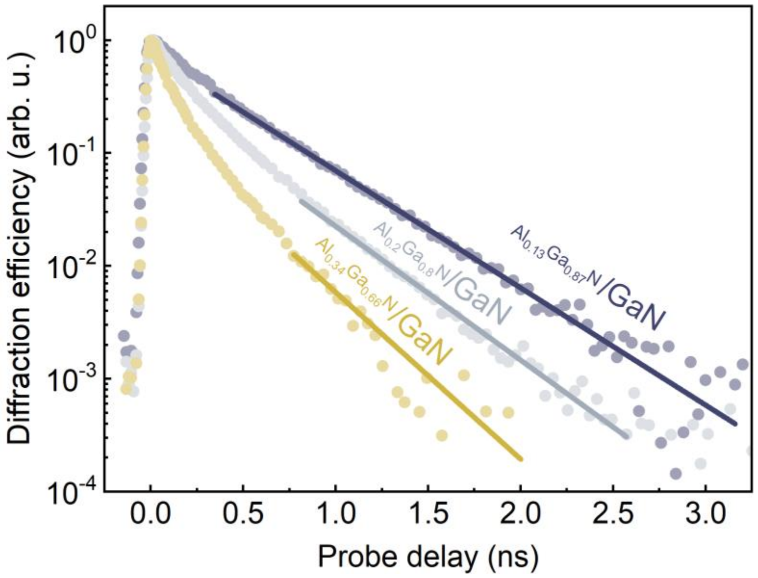

Fig IV. LITG transients for three GaN buffers (one from each sample set) recorded with 355 nm pulses at 0.3 mJ/cm² excitation.

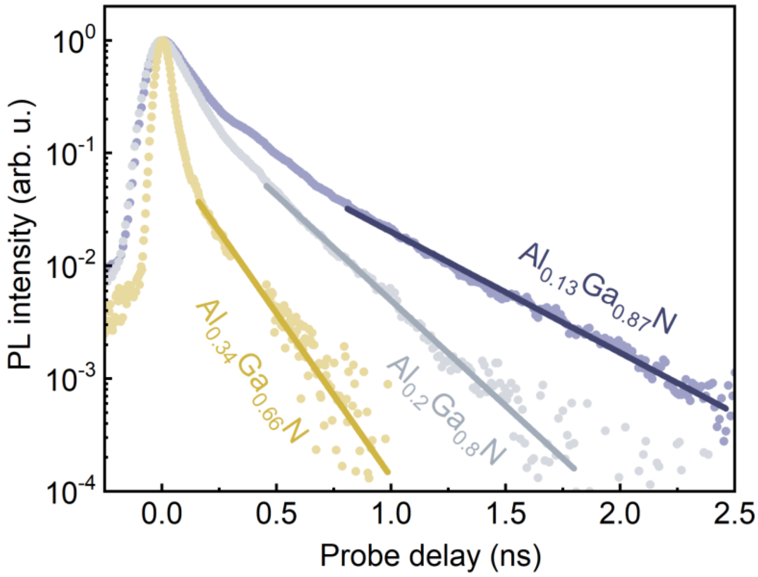

Fig V. TRPL transients for three AlGaN barriers (one from each sample set) recorded with 266 nm pulses at 0.5 mJ/cm² excitation; the transients were obtained by integrating the full spectrum of band-to-band transitions.

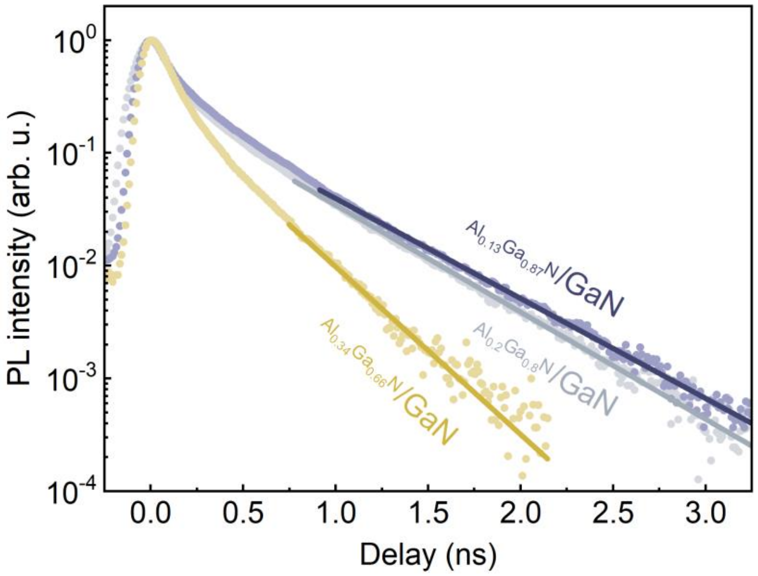

Fig VI. TRPL transients for three GaN buffers (one from each sample set) recorded with 355 nm pulses at 0.013 mJ/cm² excitation; the transients were obtained by integrating the full spectrum of band-to-band transitions.

REFERENCES:

1. Podlipskas, Ž. *et al.* Dependence of radiative and nonradiative recombination on carrier density and Al content in thick AlGaN epilayers. *J. Phys. D. Appl. Phys.* **49**, (2016).
